# Thickness Impact on the Morphology, Strain Relaxation and Defects of Diamond Heteroepitaxially Grown on Ir/Al_2_O_3_ Substrates

**DOI:** 10.3390/ma15020624

**Published:** 2022-01-14

**Authors:** Ruozheng Wang, Fang Lin, Qiang Wei, Gang Niu, Hong-Xing Wang

**Affiliations:** 1 Ministry Education Key Laboratory of Physical Electronics and Devices, School of Electronic Science and Engineering, Xi’an Jiaotong University, Xi’an 710049, China; wangrz@xjtu.edu.cn (R.W.); leaf-lin@xjtu.edu.cn (F.L.); wbgwei@mail.xjtu.edu.cn (Q.W.); 2 Key Laboratory of the Ministry of Education & International Center for Dielectric Research, School of Electronic Science and Engineering, Xi’an Jiaotong University, Xi’an 710049, China

**Keywords:** heteroepitaxial diamond, film thickness, morphology, TEM, etching pits

## Abstract

This paper investigates the formation and propagation of defects in the heteroepitaxial growth of single-crystal diamond with a thick film achieving 500 µm on Ir (001)/Al_2_O_3_ substrate. The growth of diamond follows the Volmer–Weber mode, i.e., initially shows the islands and subsequently coalesces to closed films. The films’ strain imposed by the substrate gradually relaxed as the film thickness increased. It was found that defects are mainly located at the diamond/Ir interface and are then mainly propagated along the [001] direction from the nucleation region. Etching pits along the [001] direction formed by H_2_/O_2_ plasma treatment were used to show defect distribution at the diamond/Ir/Al_2_O_3_ interface and in the diamond bulk, which revealed the reduction of etching pit density in diamond thick-film surface. These results show the evident impact of the thickness on the heteroepitaxially grown diamond films, which is of importance for various device applications.

## 1. Introduction

Diamond is a promising material for high power and high frequency electronic devices owing to its excellent material properties, e.g., ultra-wide band gap (5.5 eV), high thermal conductivity (2200 W/m·K), high breakdown voltage (10^7^ V/cm), high electron and hole mobility (4500 cm^2^/V·s and 3800 cm^2^/V·s), and low dielectric constant (5.7) [1,2,3,4,5]. Heteroepitaxially grown single-crystal diamond is considered a promising method for realizing large-area diamond substrate [6]. Diamond grown on an iridium (Ir) buffer layer has been proved good crystallinity [7,8,9]. However, due to the difference of lattice constants between diamond and Ir, the crystal lattice mismatch, strains and defects are generated from diamond/Ir interface [10,11,12,13], leading to the higher defect density of heteroepitaxial diamond compared with homoepitaxial samples [14,15,16]. Therefore, it is essential to understand the origin and propagation of defects during the different growth stages of heteroepitaxial diamond, which could make great significance for the preparation and practicability of large-area, high-quality diamond substrate.

In this work, heteroepitaxial diamond on Ir/Al_2_O_3_ substrates has been performed by MPCVD (microwave plasma chemical vapor deposition) with different thickness. Methods such as scanning electron microscope (SEM), atomic force microscope (AFM) and X-ray diffraction (XRD) are introduced to analyze the morphology and crystallinity of initial diamond growth. Then, transmission electron microscope (TEM) is used to observe the cross-section of the diamond/Ir (001) interface which is fabricated by FIB. The etching pit distribution on the surface of a heteroepitaxial diamond along the (001) direction is detected by SEM. It is clear that the etching pit density at the diamond/Ir interface is larger due to lattice mismatch. With the increase in film thickness, the diamond is coalesced to the closed film combined with strain relaxation so that the etching pit density reduces a lot. This research could provide important evidence for the understanding of the dislocation distribution of heteroepitaxial diamond thick films, both at the interface and in the film bulk.

## 2. Experimental

A 300 nm Ir (001) film was deposited on 10 mm × 10 mm Al_2_O_3_ [11,12,13,14,15,16,17,18,19,20] substrate by magnetron sputtering (ACS-4000-C4, ULVAC, Japan). The deposition power was 75 W, the Ar flow was 50 sccm, and the deposition rate was about 2 nm/min. Then, the (001)-oriented diamond was fabricated on Ir/Al_2_O_3_ substrate using bias enhanced nucleation (BEN) method by direct current chemical vapor deposition (DC-CVD, made by ourselves). The total gas flow rate was 500 sccm, the CH_4_/H_2_ flow ratio was 5%, the gas pressure was 25 torr, the direct current was 1.5 A, the temperature was 900 °C, a bias voltage on substrate of 350 V, and the duration time was 150 s [17]. After BEN, the diamond nuclei were formed on the Ir surface. Ir/Al_2_O_3_ nucleated substrates were put in MPCVD (AX5250S Seki Technotron Corp., Tokyo, Japan) for epitaxial diamond growth. The deposition power was 2500 W, the chamber pressure was 90 torr, the temperature was 950 °C, the total gas flow rate was 500 sccm, the CH_4_/H_2_ flow ratio was 5%, and the N_2_/H_2_ flow ratio was 0.03%. SEM (ZEISS, Crossbeam 540, Jena, Germany), AFM (SPI, 3800-SPA-400, Osaka, Japan) and XRD (Panalytical, X’Pert PROMRD, Almelo, The Netherlands) were used to analyze the effect of thickness on the diamond surface morphology and crystallinity. Furthermore, four samples were selected to grow 5 mins, 10 mins, 20 mins and 40 mins, respectively, which were defined as sample 1 to sample 4 (S_1_ to S_4_). Finally, after 100 h of growth, the 500 µm of diamond thick film was obtained (sample 5, S_5_).

Then, S_5_ was cut and polished along (110) plane with a roughness nearly 2 nm [18]. After that, the sample was etched by H_2_/O_2_ plasma at the gas flow of 500/5 sccm, the temperature of 900 °C, and the duration time of 30 min. Etch pits appeared where the dislocations emerged at the crystal surface [19,20]. TEM (JEOL, JEM 2100 F, Tokyo, Japan) was used to observe the interface of diamond/Ir. SEM was applied to observe the surface and cross-section of heteroepitaxial diamond. Different regions located in the cross-section of S_5_ were tested by Raman spectra (Renishaw, inVia, Banbury, UK).

## 3. Results and Discussion

**Figure 1:**
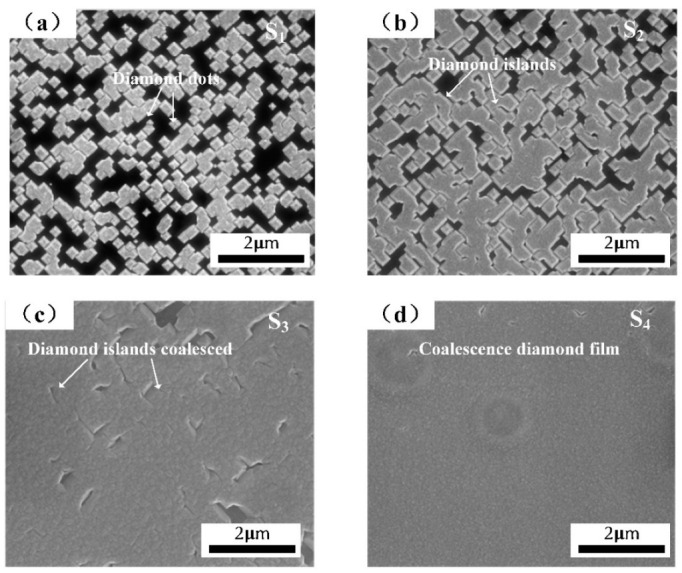
SEM observation of diamond at different growth time after nucleation. (**a**) 5 min; (**b**) 10 min; (**c**) 20 min; (**d**) 40 min.

Figure 1 showed the variation of heteroepitaxy diamond morphology observed by SEM. In Figure 1a, diamond epitaxial layers contained with a lot of highly oriented diamond dots had appeared on the Ir/Al_2_O_3_ substrate. Then, the adjacent diamond dots gradually combined to form an island grains distribution (shown in Figure 1b). Moreover, the island-shaped grains had gradually coalesced, becoming a closed thin-film structure, only few areas represented grain gaps that were not entirely coalesced (shown in Figure 1c). The diamond film finally formed a complete closed film as shown in Figure 1d, which provided a precondition for the thick-film growth of S_5_.

**Figure 2:**
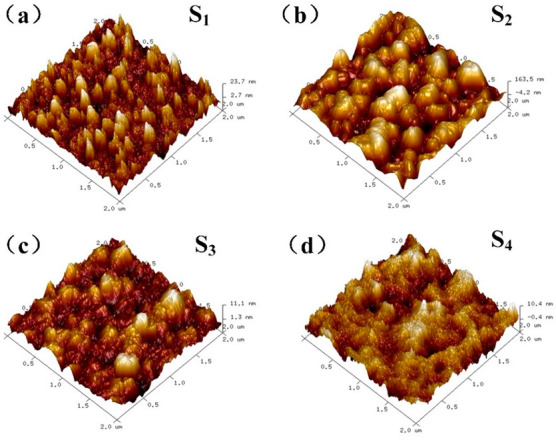
AFM observation of diamond at different growth time after nucleation. (**a**) 5 min; (**b**) 10 min; (**c**) 20 min; (**d**) 40 min.

AFM with the range of 2 µm × 2 µm was applied to observe the film roughness of S_1_ to S_4_, which is described in Figure 2. Figure 2a shows that the surface of diamond dots presented scattered square humps with a grain size of about 120 nm and thickness of about 15 nm. For the S_2_ sample, the grain size showed an obvious increase, with a grain size of about 400 nm and a thickness of about 200 nm (shown in Figure 2b), which attributed to island growth mode. Then, the image of S_3_ showed that the fluctuation of the film decreased rapidly, and the height steps reduced to about 10nm, representing the characteristics of closed diamond films (shown in Figure 2c). Lastly, in Figure 2d, the flatness of diamond films was achieved, indicating that the diamond grains have coalesced and have formed a closed film. The root mean square (RMS) roughness of four samples were 4.44, 41.1, 2.05 and 2.37 nm, respectively, reflecting the variation of film morphology during initial diamond growth. We guessed that increase of S_4_ could possibly be due to the selection of test area, e.g., substrate roughness, non-coalesced film, or impurities adsorbed on the film surface.

**Figure 3:**
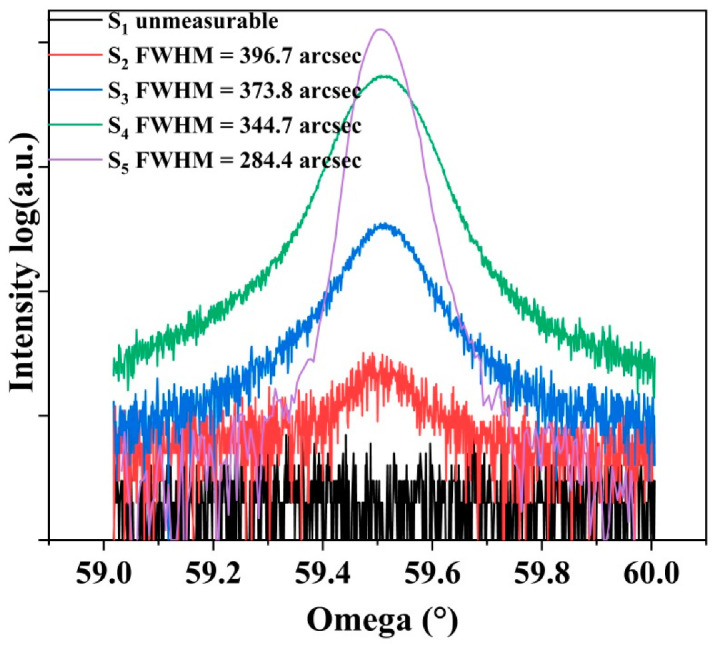
XRD rocking curves of heteroepitaxial diamond at different growth stages after nucleation (S_1_ to S_4_) and the thick film (S_5_).

Figure 3 represented the XRD rocking curves of diamond (004) orientation at different growth stages. Obviously, there was no diamond (004) characteristics peak in the initial growth stage when heteroepitaxial diamond film is too thin to form a continuous and dense film (corresponded to S_1_). Then, with the increase in film thickness, the diamond (004) peak gradually appeared and grew in intensity. Meanwhile, as growth continues, the full widths at half maximum (FWHM) of (004) rocking curves were decreased, at which the FWHM of S_5_ measured in the (004) direction was 284.4 arcsec, showing a decent crystallinity of heteroepitaxy diamond grown on Ir/Al_2_O_3_ substrate.

**Figure 4:**
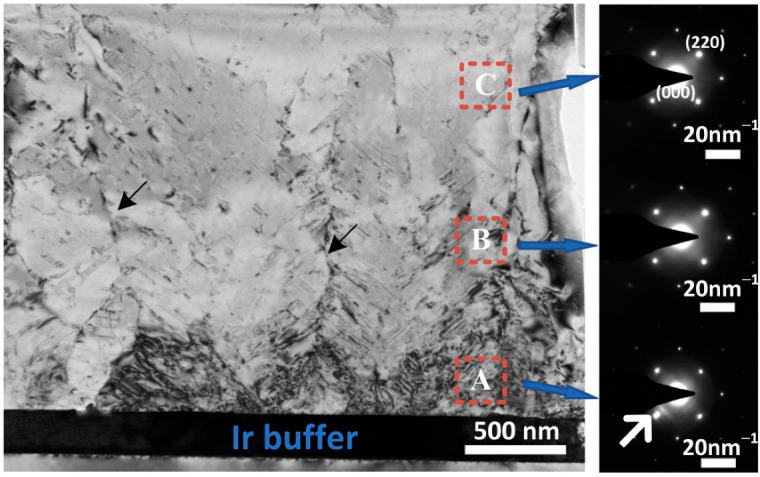
TEM image of heteroepitaxy diamond on Ir (001)/Al_2_O_3_ substrate along [110] zone axis (area A: near diamond/Ir interface; area B: above the diamond/Ir interface; area C: in the diamond bulk.).

As mentioned above, in order to study the propagation of defects in heteroepitaxial diamond, a thick diamond film (S_5_, 500 µm) was deposited by MPCVD. TEM was adopted to characterize the cross-section of diamond bulk along [110] zone axis (ZA). The propagation of defects at the diamond/Ir interface was directly observed by a TED mode at 400 kV. The sample (10 µm × 5 µm × 100 nm) was prepared on the back of utilizing a focused ion beam (FIB) technology. The bright diamond epitaxial layer and the dark Ir metal layer could be detected in the Figure 4, as well as noticeable dark stripes in the diamond bulk. These shadows revealed that the diamonds gradually converted from single grains into coalesced films, as shown in area A in Figure 4. Especially in the regions above Ir layer, there were lots of dislocations with an angle of 45° along the Ir (001) plane. With the increase in film thickness, diamond grains gradually coalesced, along with dislocations extended to the surface (shown in area B in Figure 4), corresponding to the image that is displayed in Figure 1d. Selected area diffraction pattern (SADP) images were measured in order to make sure the crystal orientation of the TEM images. The results showed that in the area A near diamond/Ir interface, there were regular spots representing cubic crystal systems in the electron diffraction spots. After the calculation of SADP images, the crystal plane spacing of diamond (2–20) plane was 1.274 Å, increased by 1% compared to standard diamond card (1.261 Å), which was related to the epitaxial diamond growth from Ir/Al_2_O_3_ substrate. In addition, the crystal-plane spacing of Ir (2–20) was larger than that of diamond (1.357 Å), and two azimuthally broadened diffraction spots can be observed at the SADP of area A, indicating the in-plane disorientation of the initial diamond islands grown on the Ir layer. This means, because of the small disorientation of the Ir buffer, the diamond islands at the very beginning of the growth had a tendency to be textured [21]. As growth continued, such a tendency stopped and the diamond layer followed the single crystalline epitaxial growth. Meanwhile, the asymmetric diffraction spots disappeared in the SADP images at area B and C, and the lattice constants were 1.267 Å and 1.265 Å, respectively, which were close to the intrinsic diamond.

**Figure 5:**
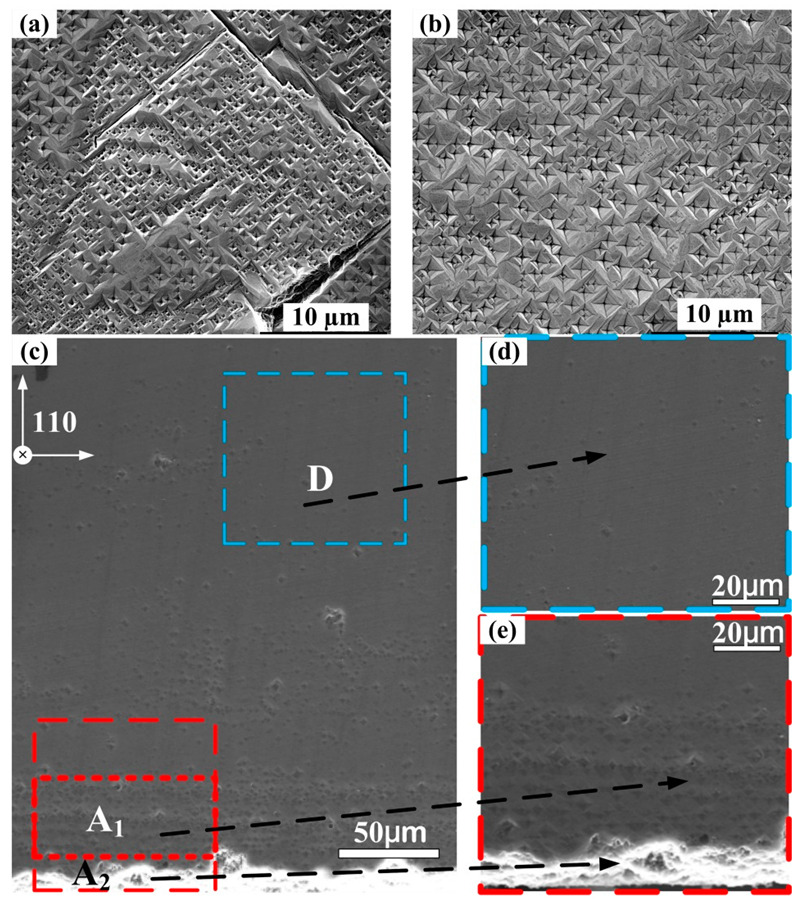
Etching pits of the heteroepitaxial diamond along [001] direction (**a**) at diamond/Ir interface and (**b**) at the surface of diamond thick film; Etching pits of heteroepitaxial diamond cross-sections along [220] direction (**c**) the film bulk distributions. (**d**) the magnification at diamond/Ir interface. (**e**) the magnification near film surface. (A_1_: 20 µm above diamond/Ir interface. A_2_: at the diamond/Ir interface; D: 200 µm above diamond/Ir interface).

Additionally, the morphology of heteroepitaxial diamond after H_2_/O_2_ plasma treatment was observed by SEM. Figure 5a,b showed the etching pit distributions both at the diamond/Ir interface and the film surface along the (001) direction, which represented extensive overlapping rectangles appearance. Some cracks were found in Figure 5a which was attributed to the strain between Al_2_O_3_ substrate and diamond thin film [22], but it has no relationship with the defects since the cracks were formed before H_2_/O_2_ plasma treatment. Moreover, the lower etching pit density was observed on Figure 5b, indicating partial dislocations were annihilated in the epitaxial layer during diamond growth. The shape of etching pits depended on the angle of dislocation terminated at the diamond/Ir surface which could be observed at Figure 1a before [23], illustrating that the propagation of dislocations were mainly along (001) from the nucleation region [24].

Furthermore, etching pits distribution in the cross-section region extending from the interface to diamond bulk was shown in Figure 5c–e. At the diamond/Ir interface, numerous etching pits and rough epitaxial layers were detected, representing the poor crystal quality at grain boundaries (area A_2_) [24]. Then, as the initial growth continued, especially in the film bulk of 20 µm above diamond/Ir interface (area A_1_), a relatively high etching pit density along the (220) direction was obtained. When the thickness of the diamond film exceeded 200 µm, etching pit density reduced notably due to the improved epitaxial crystallinity (area D). It was clear that the etching pits were not continuously distributed in the whole diamond bulks. We could infer that due to the larger lattice mismatch at diamond/Ir/Al_2_O_3_ substrate, a great quantity of etching pits were located at interface. During the growth mode of diamond transition from dispersed islands to coalescence film (shown in Figure 1), the etching rate by H_2_/O_2_ plasma treatment on the film was also changed. Therefore, the heteroepitaxial diamond film had undergone a relaxation process. With the increase in film thickness, the lattice constant of the epitaxial film was close to the diamond (220) standard value (1.265 Å vs. 1.261 Å), and the strain of the film was fully relaxed. The epitaxial layer was formed as high-quality and dense film.

**Figure 6:**
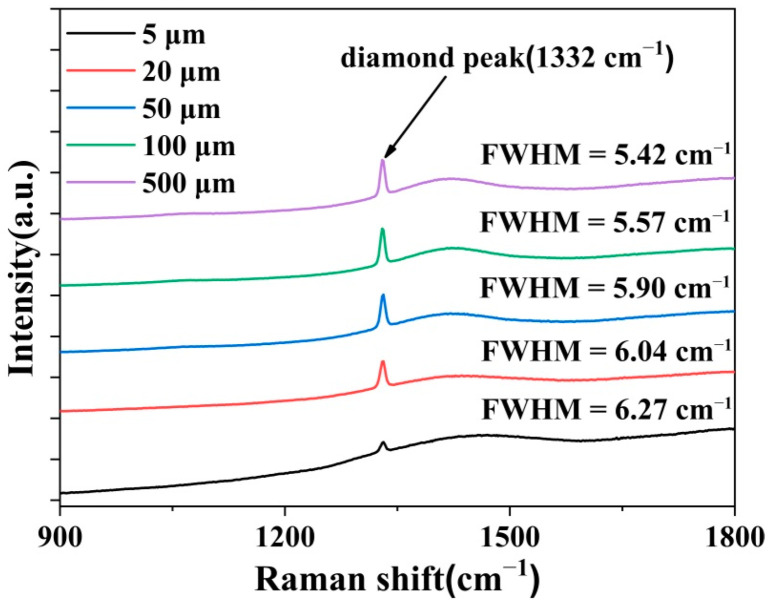
Raman spectra at different diamond cross-section positions above the diamond/Ir interface.

Then, a Raman spectrum was used to test the diamond characteristics peaks and defects at different positions of the diamond bulk, which is shown in Figure 6. The laser wavelength was 532 nm, and we chose five positions on the cross-section of the diamond bulk, which were 5, 20, 50, 100, 500 µm away from the diamond/Ir interface, respectively. A weak first-order diamond characteristic peak (1332 cm^−1^) could be observed at the position of 5 µm above the diamond/Ir interface. However, there were fluorescence peaks related to nitrogen (N) defects in a wide range of 1460 cm^−1^, the intensity of which exceeded that of the diamond characteristic peaks [25,26]. As growth continued, the intensity of the diamond characteristic peak significantly increased, while the nitrogen-related peaks remained stable. This phenomenon depicted that there were a large number of N vacancies located in grain boundaries during the initial growth of the diamond. Then, the grains coalesced to closed films so that the N vacancy concentrations kept stable [27,28]. Last, the Raman peak width was reduced from 6.27 cm^−1^ to 5.42 cm^−1^ with the increase in film thickness, indicating the better crystal quality and lower defects at the heteroepitaxial diamond film surface.

## 4. Conclusions

In summary, the impact of film thickness on heteroepitaxially grown diamond is explored. SEM results show the evolution of diamond morphology from dots, islands, and then coalesced to closed film. AFM results display that the island growth possesses the largest roughness of 41.1 nm, and then a smooth surface with RMS of 2.37 nm is obtained. XRD rocking curves depict that the decent FWHM of 284.4 arcsec is obtained from the diamond thick film. The dislocations distribution in heteroepitaxial diamond extending from interface to the film bulk is observed by TEM and SEM. Numerous etching pits near the interface owing to the small disorientation of the initial diamond islands grown on the Ir layer. With the coalescence of diamond islands to closed film, the diamond bulk strain is relaxed so that the dislocations are reduced. This study illustrates the generation and evolution of dislocation in the whole heteroepitaxial diamond thick film, which will contribute to promote the quality of heteroepitaxial diamond substrate and electronic devices.

## Data Availability

Data available on request due to restrictions, e.g., privacy or ethical.
